# Effects of 445 nm, 520 nm, and 638 nm Laser Irradiation on the Dermal Cells

**DOI:** 10.3390/ijms222111605

**Published:** 2021-10-27

**Authors:** Łukasz Szymański, Martyna Ciepielak, Aleksandra Cios, Małgorzata Palusińska, Wanda Stankiewicz, Sławomir Lewicki

**Affiliations:** 1 Department of Molecular Biology, Institute of Genetics and Animal Biotechnology, Polish Academy of Science, Postępu 36A, 05-552 Magdalenka, Poland; m.palusinska@igbzpan.pl (M.P.); s.lewicki@igbzpan.pl (S.L.); 2 Department of Microwave Safety, Military Institute of Hygiene and Epidemiology, 01-163 Warsaw, Poland; ciepielakmartyna@gmail.com (M.C.); aleksandracios@gmail.com (A.C.); wanda.stankiewicz@gmail.com (W.S.); 3 Faculty of Medical Sciences and Health Sciences, Kazimierz Pulaski University of Technology and Humanities, 26-600 Radom, Poland

**Keywords:** laser irradiation, skin exposition, dermal effect of laser, 445 nm, 520 nm, 638 nm, Ker-CT, BJ-5ta, proliferation, cell cycle, viability

## Abstract

**Background:** The invention of non-ionizing emission devices revolutionized science, medicine, industry, and the military. Currently, different laser systems are commonly used, generating the potential threat of excessive radiation exposure, which can lead to adverse health effects. Skin is the organ most exposed to laser irradiation; therefore, this study aims to evaluate the effects of 445 nm, 520 nm, and 638 nm non-ionizing irradiation on keratinocytes and fibroblasts. **Methods:** Keratinocytes and fibroblasts were exposed to a different fluency of 445 nm, 520 nm, and 638 nm laser irradiation. In addition, viability, type of cell death, cell cycle distribution, and proliferation rates were investigated. **Results:** The 445 nm irradiation was cytotoxic to BJ-5ta (≥58.7 J/cm^2^) but not to Ker-CT cells. Exposure influenced the cell cycle distribution of Ker-CT (≥61.2 J/cm^2^) and BJ-5ta (≥27.6 J/cm^2^) cells, as well as the Bj-5ta proliferation rate (≥50.5 J/cm^2^). The 520 nm irradiation was cytotoxic to BJ-5ta (≥468.4 J/cm^2^) and Ker-CT (≥385.7 J/cm^2^) cells. Cell cycle distribution (≥27.6 J/cm^2^) of Ker-CT cells was also affected. The 638 nm irradiation was cytotoxic to BJ-5ta and Ker-CT cells (≥151.5 J/cm^2^). The proliferation rate and cell cycle distribution of BJ-5ta (≥192.9 J/cm^2^) and Ker-CT (13.8 and 41.3 J/cm^2^) cells were also affected. **Conclusions:** At high fluences, 455 nm, 520 nm, and 638 nm irradiation, representing blue, green, and red light spectra, are hazardous to keratinocytes and fibroblasts. However, laser irradiation may benefit the cells at low fluences by modulating the cell cycle and proliferation rate.

## 1. Introduction

Lasers are a widely used tool in industry, communication, science, medicine, and the military [1]. These monochromatic light sources have allowed for the development of many advanced technologies such as superresolution imaging or optical tweezers. In medicine, the applications include diagnostics, medical treatments, aesthetic medicine corrections and many more [2,3,4,5]. Currently, there are 1204 active clinical trials using lasers in medical applications. In addition, countless other applications of lasers, not directly linked to medicine, are developed worldwide. However, with the widespread presence of laser-employing technology, there is an increased risk of accidental exposure to non-ionizing irradiation. Among others, skin, which is the largest human organ, may be indirectly exposed to laser radiation through the reflection of the beam from different surfaces.

**Figure 1:**
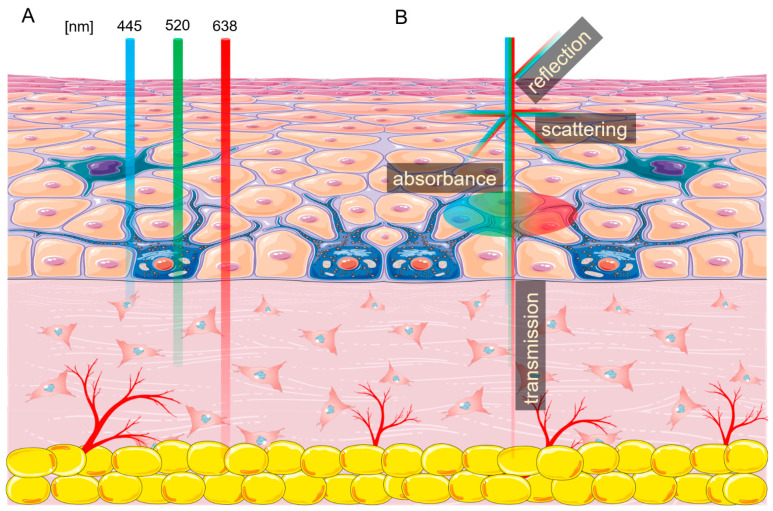
Laser interactions with skin. (**A**) Skin penetration by different wavelengths of light. (**B**) Laser beam interaction with the skin. The figure was created using SMART (Servier Medical ART) modified graphics, licensed under a Creative Commons Attribution 3.0. Generic License.

As described previously, the main difference of laser light that distinguishes it from other light sources is the consistency and coherency of the beam [1,6]. However, lasers have other key characteristics, with the most important being the wavelength, which is positively associated with the penetration depth and thermal effect [7]. The wavelength-skin penetration depth relationship is illustrated in Figure 1A. Another important laser characteristic is the fluence specified by the laser energy and the duration of radiation. Fluence determines the irradiation dose absorbed by the cells, affecting the cell’s metabolism [8]. Finally, the biological effect of laser radiation depends on the type of the impulse —pulsating or continuous.

Every tissue exposed to non-ionizing radiation generated by lasers interacts with it in four ways—absorption, scattering, reflection, and transmission (Figure 1B) [9]. As light enters the skin, about 4–7% is reflected, and some parts are scattered in a different direction [10]. The laser energy which passes through the tissue is called transmission. Finally, the photon energy accepted by the cell is called absorption. This energy can be reemitted or transformed into heat, increasing tissue temperature [11,12,13]. The amount of energy absorbed by the tissue depends on the presence of different chromophores, with each absorbing a specific wavelength or wavelengths. The three main endogenous chromophores in the skin are melanin, water, and hemoglobin [6,14]. Many chromophores can be affected by more than one wavelength of light, such as hemoglobin, which has three absorption picks—415 nm, 540 nm, and 577 nm. In addition, the photons absorbed by the chromophore can cause photoacoustic (mechanical), thermal, or chemical changes.

Over the years, a number of laboratories have demonstrated that non-ionizing radiation can change cells’ metabolism and affect proliferation, differentiation, cell cycle, and cell division [15]. Laser radiation can also alter RNA and DNA synthesis as well as DNA repair pathways [16,17]. On the tissue level, lasers have been shown to affect inflammation and modulate the immune response [18]. Most of those properties were carefully investigated and employed to provide clinical benefits in various scenarios. However, it is essential to remember that the laser-cell interactions are wavelength, dosage, and cell type-specific.

Consequently, accidental expositions may result in adverse health effects [19,20]. Therefore, in this study, we used a laser exposure system to investigate the effects of 445 nm, 520 nm, and 638 nm laser irradiation on fibroblast and keratinocyte cell viability, proliferation, and cell cycle. Keratinocyte and fibroblast cell cultures were chosen for this research as they are the main cellular components of the skin. Specifically, keratinocytes constitute approximately 90–95% of cells in the epidermis, while fibroblasts are the principal cells of the dermis [21,22].

## 2. Results

### 2.1. Biological Effects of 455 nm Laser Radiation

*Fibroblasts*. BJ-5ta viability of exposed cells decreased significantly from the fluence of 59.7 J/cm^2^ up to 91.8 J/cm^2^. The loss of viability was dose-dependent. The relative proliferation of BJ-5ta fibroblasts also decreased significantly, starting at the fluence of 50.5 J/cm^2^. Differences were also observed in the cell cycle distribution. The percentage of G1 cells decreased, and the percentage of G2/M increased starting at the fluence as low as 27.6 J/cm^2^. Again, the changes were dose-dependent except from the cells exposed to 59.7 J/cm^2^, for which a statistically significant increase of cell population in the S phase was observed instead of G2/M.

**Figure 2:**
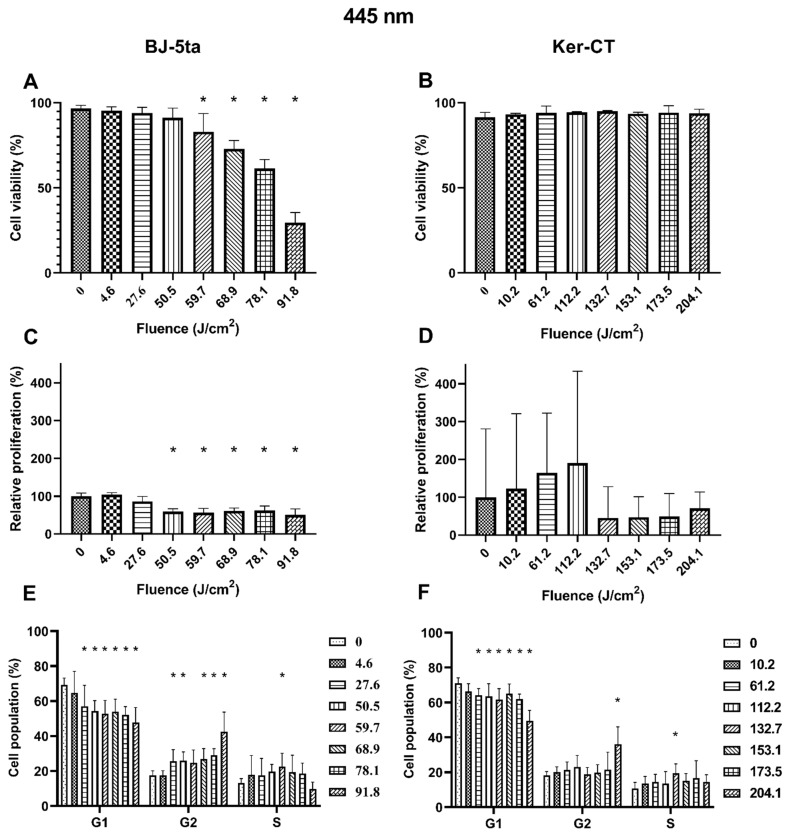
Effects of 445 nm irradiation on BJ-5ta fibroblasts and Ker-CT keratinocytes. (**A**,**B**) Viability using Annexin V-APC and PI. (**C**,**D**) Relative proliferation using an anti-Ki67-APC antibody. (**E**,**F**) Cell cycle distribution using FxCycle PI/RNase Staining Solution. * *p* < 0.05.

*Keratinocytes*. On the other hand, the 445 nm laser irradiation did not affect the viability of the Ker-Ct keratinocytes. There were no significant differences in relative proliferation rates even though changes in the cell cycle phase distribution were observed. The percentage of cells in the G1 phase decreased when exposed to at least 61.2 J/cm^2^. The population of cells in the G2/M phase was increased only in cells exposed to 204.1 J/cm^2^. Additionally, the cells exposed to a fluence of 132.7 J/cm^2^ are characterized by a percentage increase of cells in the S phase. Results of the effects of 445 nm laser irradiation on fibroblasts and keratinocytes are presented in Figure 2.

### 2.2. Biological Effects of 520 nm Laser Radiation 

*Fibroblasts*. The 520 nm laser radiation affected BJ-5ta cells’ viability only at the fluence of 468.4 J/cm^2^. In addition, exposure to the 520 nm laser did not affect the BJ-5ta fibroblast proliferation rate or cell cycle distribution.

**Figure 3:**
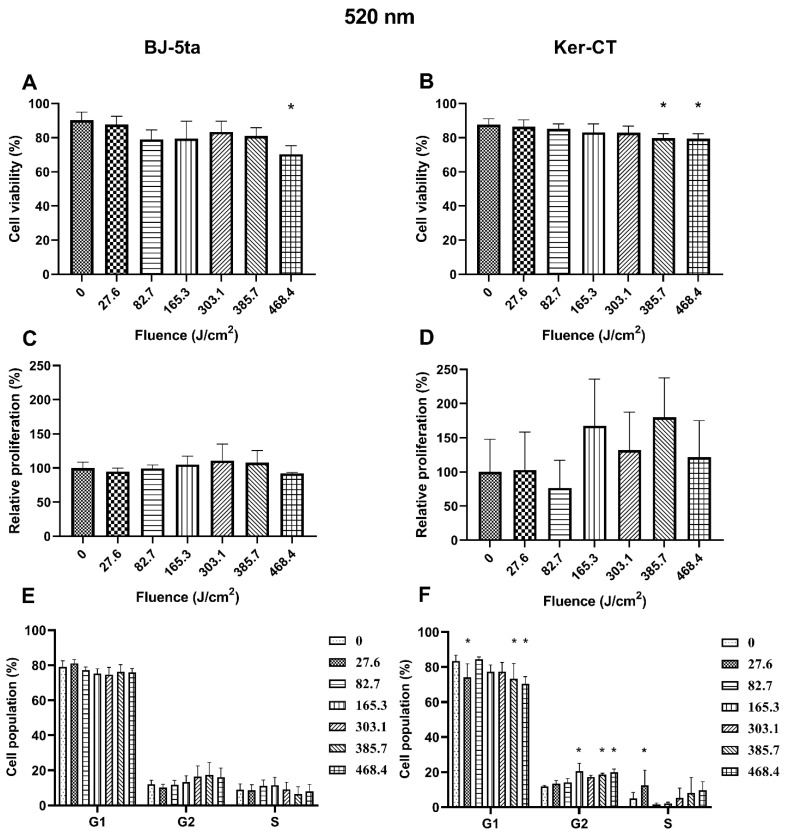
Effects of 520 nm irradiation on BJ-5ta fibroblasts and Ker-CT keratinocytes. (**A**,**B**) Viability using Annexin V-APC and PI. (**C**,**D**) Relative proliferation using an anti-Ki67-APC antibody. (**E**,**F**) Cell cycle distribution using FxCycle PI/RNase Staining Solution. * *p* < 0.05.

*Keratinocytes*. In Ker-CT keratinocytes, we observed a decrease in cell viability starting at the fluence of 385.7 J/cm^2^. No significant changes in the proliferation rate were observed. Exposure to 520 nm laser irradiation caused a decrease in the percentage of cells in the G1 phase at fluence 27.6, 385.7, and 468.4 J/cm^2^. We also observed a significant increase of cells in the G2/M phase (165.3, 385.7, and 468.4 J/cm^2^) and the S phase (27.6 J/cm^2^). Effects of exposure of keratinocytes and fibroblasts to 520 nm laser irradiation are presented in Figure 3.

### 2.3. Biological Effects of 638 nm Laser Radiation

*Fibroblasts*. Exposure to 638 nm laser irradiation resulted in decreased viability of BJ-5ta cells starting at the fluence of 151.5 J/cm^2^. Exposure to 192.9 J/cm^2^ and 234.2 J/cm^2^ of the 620 nm laser also reduced the relative proliferation rate of fibroblasts. Cell cycle distribution was also affected by the 638 nm laser. The percentage of BJ-5ta cells in the S phase decreased when exposed to 192.9 J/cm^2^ and 234.2 J/cm^2,^ compared to the sham exposed control.

**Figure 4:**
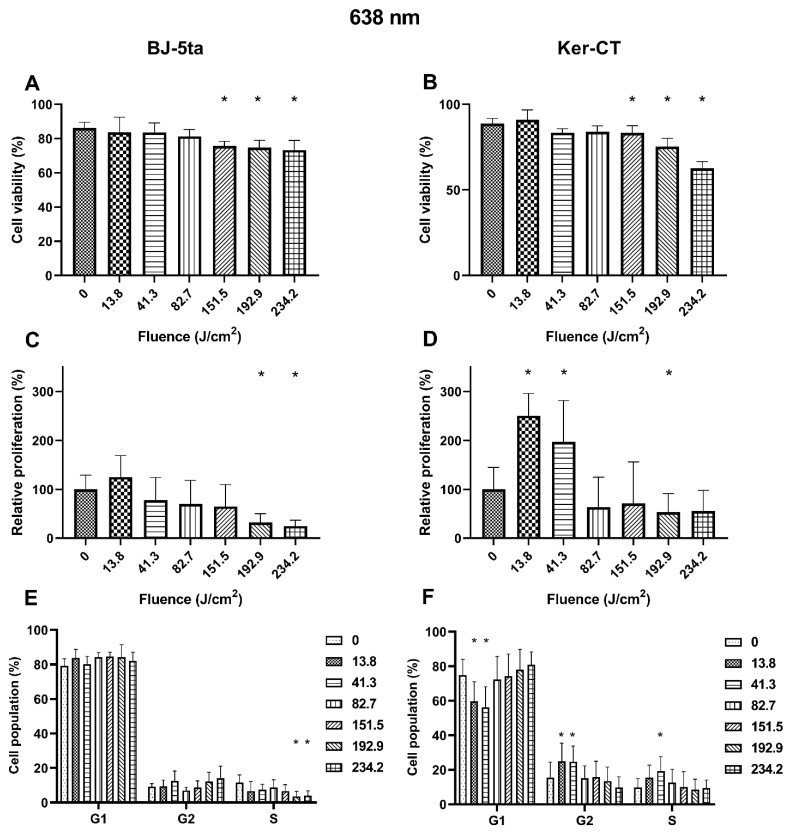
Effects of 638 nm irradiation on BJ-5ta fibroblasts and Ker-CT keratinocytes. (**A**,**B**) Viability using Annexin V-APC and PI. (**C**,**D**) Relative proliferation using an anti-Ki67-APC antibody. (**E**,**F**) Cell cycle distribution using FxCycle PI/RNase Staining Solution. * *p* < 0.05.

*Keratinocytes*. Likewise, the Ker-CT viability also decreased after exposure to at least 151.5 J/cm^2^. Moreover, we observed an increase in the proliferation rate of keratinocytes exposed to 13.8 and 41.3 J/cm^2^ of the 638 nm laser and a decrease when exposed to the higher energy of 192.9 J/cm^2^. In addition, cell cycle analysis revealed a significant decrease of cells in the G1 phase (13.8 and 41.3 J/cm^2^) associated with the increase of cells in the G2/M phase (13.8 and 41.3 J/cm^2^) and the S phase (41.3 J/cm^2^). Effects of the exposure of keratinocytes and fibroblasts to 638 nm laser irradiation are presented in Figure 4.

## 3. Discussion

The 445 nm irradiation represents blue light spectra. In our study, we observed reduced viability of BJ-5ta fibroblast when exposed to at least 59.7 J/cm^2^. The loss of viability was caused mainly by apoptosis which was a roughly two-fold more common death type than necrosis (Supplementary Data). These data support the results obtained by Mignon et al., who observed increased cytotoxicity of cells exposed to 450 nm [23]. The dose-dependent decrease in viability is most likely associated with the increased ROS generation by cells exposed to blue light [23,24]. The Ki-67 protein is a cellular marker of proliferation that is present during all active phases of the cell cycle (G1, S, G2, and mitosis) but is absent in resting cells (G0) [25]. In addition, the cellular content of the Ki-67 increases as the cell progresses through the S phase of the cell cycle [26]. Based on the Ki67 results, it was found that exposition to at least 50.5 J/cm^2^ of blue light (445 nm) inhibits the cell proliferation capabilities of fibroblasts. Our findings confirm the results obtained by Oplander et al. and Mamalis et al., who also showed inhibiting properties of blue light on fibroblast proliferation [24,27]. The influence of the 445 nm laser is also reflected in the changes in the cell cycle distribution starting at the fluence of 27.6 J/cm^2^. An increased percentage of cells in the G2/M phase slowed cell growth and therefore decreased the proliferation rate.

On the other hand, neither the viability nor the proliferation rate of Ker-CT keratinocytes changed after being exposed to 445 nm blue light. However, one can observe a not significantly increasing proliferation tendency in keratinocytes exposed to 445 nm irradiation up to 112.2 J/cm^2^. The tendency is the opposite for keratinocytes exposed to higher energy fluence. The 445 nm blue light also influenced the cell cycle distribution when compared to the control. The percentage of cells in G1 decreased while the percentage of S and G2/M increased. Keratinocytes during their life cycle either differentiate or divide and proliferate. As the population differentiates, the number of cells in the G1 phase decreases, and the number of G2/M cells increases. The process is also characterized by a transient increase of S phase cells and the presence of polyploids [28,29]. The observed changes in the cell cycle are consistent with the keratinocyte differentiation process, indicating that the 445 nm irradiation may induce differentiation. The results are in line with findings by Liebmann et al., who showed that the 453 nm irradiation could induce differentiation of skin cells by a generation of nitric oxide from nitrosated proteins [30].

The exposition of BJ-5ta cells to 520 nm irradiation, which represents green light spectra, decreased the viability of the cells only at the maximal fluence of 468.4 J/cm^2^. The decrease in viability was caused by necrosis (Supplementary Data), which was probably caused by the thermal effect, as shown by Jurczyszyn et al. [31]. The green light exposition up to 468.4 J/cm^2^ did not affect the cells in any other way. More effects of 520 nm radiation were observed in keratinocytes. KerCT cells exposed to at least 385.7 J/cm^2^ of 520 nm irradiation were characterized by decreased viability. The death of the cells was caused equally by apoptosis and necrosis (Supplementary Data). The changes were also observed in cell cycle distribution. The low fluence of 27.6 J/cm^2^ favored the S phase, while a high energy fluence of 385.7 and 468.4 J/cm^2^ induced an increase in the percentage of cells in G2/M and concomitant reduction in the G1 phase, consistent with a G2/M arrest. During the cell cycle, there are three major checkpoints. One of them, called the spindle checkpoint, during which the attachment of sister chromatids is verified, is known to be temperature-dependent [32]. The spindle assembly is impaired at higher temperatures, and the cell cannot proceed with mitosis [33]. The thermal effect of high fluence laser irradiation probably caused the G2/M arrest observed in the present study.

The exposition of fibroblasts to 638 nm red light resulted in a significant reduction in the proportion of viable cells at the fluence of 151.5, 192.9, and 234.2 J/cm^2^ compared to the control. Notably, an increase in the percentage of necrotic but not apoptotic cells suggests that the generated thermal effect causes the cells’ death. Moreover, at the fluence of 192.9 and 234.2 J/cm^2^, we observed a reduced proliferation rate reflected by the percentage decrease of cells in the S phase. Thus, the cell cycle and proliferation rate results are consistent with cell responses to heat stress, and necrosis of the cells was caused by the excessive heat that was unmanageable for heat shock proteins (extreme hypothermia) [34,35]. In keratinocytes exposed to high fluences of 638 nm red light irradiation, we also observed decreased viability and proliferation rates compared to sham exposed cells. Again, the observed changes are consistent with the effects of heat stress [36]. However, low fluences (13.8 and 41.3 J/cm^2^) of 638 nm irradiation significantly induced the proliferation rate of Ker-CT cells. The increased proliferation rate was associated with a decrease of the G1 phase of the cell cycle with a simultaneous increase of the G2/M and S phases. The data confirm the results of other research groups. Sperandio et al. showed that a 660 nm laser enhances the proliferation of HaCaT cells at energy densities of 3, 6, and 12 J/cm^2^ [37]. Lee et al. also showed that 6.6 J/cm^2^ 630 nm irradiation accelerates keratinocyte proliferation by promoting DNA synthesis in the epidermis [38].

The result shows that Ker-CT keratinocytes are more resistant to 445 nm irradiation than BJ-5ta fibroblasts. On the other hand, the Ker-CT cells are more sensitive to 520 nm irradiation than fibroblasts. Finally, the 638 nm irradiation seems beneficial for keratinocytes at low fluences and is harmful to BJ-5ta and Ker-CT cells at high fluences. The result showed that, indeed, the laser-cell interactions are wavelength, dosage, and cell type-specific. Different responses of cells to laser exposition may arise from the physiological localization of the cells and the tissue penetration depth, which is specific for each irradiation wavelength. Keratinocytes, found in the skin epidermis, are the first line of defense against external hazards and thus might be better evolutionary prepared to resist 445 nm irradiation characterized by a small penetration depth than fibroblasts that are rarely exposed to this wavelength. To test this hypothesis, however, further studies using 3D full-thickness skin models are necessary. Finally, care should be taken to avoid overexposure to non-ionizing radiation. Repeated laser treatments may be harmful not only to patients but also to professionals exposed to residual radiation as they usually perform multiple irradiations during a single day. Laser safety is even more critical in an industrial and military setting where high-energy lasers are employed, and the energy of the reflected beam might be significantly higher. Currently, under European IEC 60825 and US ANSI Z136 standards, the safety measures for class 4 lasers (>500 mW) focus on direct and indirect eye injury as well as direct skin burns but do not consider the effects of accidental exposure on the skin. Our data, however, suggest that exposure to laser radiation affects the skin by influencing the cell cycle, proliferation, and apoptosis without causing skin damage. Based on the cell viability, the safe fluences for each irradiation wavelength are summarized in Table 1.

## 4. Materials and Methods

Reagents were purchased from ThermoFisher Scientific, Poland, unless stated otherwise.

### 4.1. Cell Culture

KerCT and BJ-5ta acquired from the ATCC collection were used in the research. Both cell lines are hTERT-immortalized. The BJ-5ta were cultured in a 4:1 mixture of DMEM and Medium 199 with the addition of 10% FBS, 50 U/mL of penicillin, 50 µg/mL of streptomycin, and 0.01 mg/mL hygromycin B. The KerCT were cultured in a KGM-Gold medium with supplements and 50 U/mL of penicillin, and 50 µg/mL of streptomycin. The cells were cultured under standard aseptic conditions (37 °C, 95% humidity, 5% CO_2_). The cells used for the experiments (following between 3 and 18 passages) were grown in continuous cultures and were passaged after reaching 80–90% confluence with TrypLE. After thawing from the cell bank, the cells were passaged at least twice before experiments. After trypsinization, the cells were centrifuged at 400× *g* for 5 min. Finally, the cell pellets were resuspended in 1 mL of fresh media and counted with EVE Plus Automated Cell Counter (NanoEntek, Seoul, Korea).

### 4.2. Laser Exposure System

**Figure 5:**
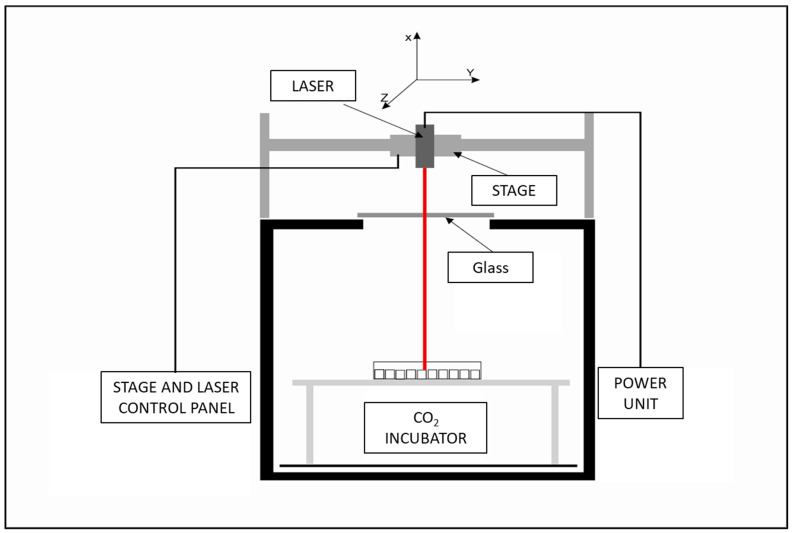
Laser exposure system schematics.

The laser exposure system developed by the Military Institute of Hygiene and Epidemiology in Warsaw allows for non-ionizing radiation exposure of cells, while other parameters crucial for cell culture, including CO_2_, temperature, and humidity, remain constant. The system is calibrated with 96-well cell culture plates, allowing for precise laser power and exposure time control. The 445 nm “blue”, 520 nm “green”, and 638 nm “red” diode lasers were purchased from Opt Lasers, Białystok, Poland. The 7.5 supply voltage powers each laser. The schematic of the laser exposure system is presented in Figure 5 and basic information about used lasers is presented in Table 2.

Effective power was measured in cell culture plate wells using a radiometer (Spectra Laser, Białystok, Poland).

### 4.3. Laser Exposure

Cells were trypsinized, counted, and seeded in a 96-well plate at a concentration of 1 × 10^5^/mL for fibroblasts and 1.5 × 10^5^/mL for keratinocytes. After 24 h, the cells were exposed to laser irradiation with the above-described laser exposure system. First, each row of the cell culture plate was exposed to the laser beam for 0–170 s. Then, cell culture plates were incubated for 24 h. After incubation, cells were trypsinized and centrifuged (350× *g*, 5 min). Then, samples were evaluated for viability, proliferation rate, and potential changes in the cell cycle. Results were presented as the mean SD of at least three independent experiments.

### 4.4. Cell Viability

Cell viability was assessed according to the procedure described elsewhere [39]. Briefly, the cell pellets were washed and resuspended in 50 μL of PBS and 4.5 μM/sample allophycocyanin (APC) conjugated annexin V (BD Bioscience, Warsaw, Poland) with 1 μg/sample of propidium iodide (PI, BD Bioscience, Warsaw, Poland). After 15 min of incubation at 4 °C, the cells were analyzed by flow cytometry (CytoFlex, Becman Culter, Warsaw, Poland). The acquisition was stopped after 10,000 cells. Finally, the percentages of live cells were analyzed using Kaluza (version 2.1. Beckman Coulter, Warsaw, Poland) and presented as the mean SD of at least three independent experiments (*n* ≥ 9).

### 4.5. Proliferation (Ki67 Assay) and Cell Cycle

After centrifugation, samples were washed twice with PBS. The cells were then resuspended in 200 µL of cold 70% ethanol (4 °C) and stored in a freezer (−20 °C) prior to analysis (1−30 days). On the analysis day, 100 µL of PBS containing 2% FBS and 1 mM EDTA was added to the cells, and samples were centrifuged (500× *g*, 5 min). Ki67 staining was performed (according to the procedure recommended by the manufacturer), and PI staining followed the procedure described in [40]. Briefly, 50 µL of PBS containing 2% FBS, 1 mM EDTA, and 5 µL of Ki67-APC antibody was added to each sample and incubated for 30 min RT in the dark. Next, the samples were washed with PBS containing 2% FBS and 1 mM EDTA, and 50 µL of the FxCycle PI/RNase Staining Solution was added for 30 min. The acquisition was stopped after 10,000 cells. The results were analyzed using Kaluza (Beckman Coulter, Poland) and presented as the mean SD of at least three independent experiments (*n* ≥ 9).

### 4.6. Statistical Analysis

All results were presented as the mean ± standard deviation (SD). The data distribution was evaluated using the Shapiro–Wilk test. All irradiated groups were compared to a sham irradiated control. Statistical evaluation of viability and the proliferation rate was performed using the Kruskal–Wallis test with Dunn’s multiple comparison test. Finally, cell cycle results were analyzed using 2way ANOVA with Šídák’s multiple comparisons test. GraphPad Prism software (version 9.2.0; GraphPad Software, Inc., La Jolla, CA, USA) was used for all evaluations. *p* < 0.05 was considered statistically significant.

### 4.7. Calculations

BJ-5ta fibroblasts and Ker-CT keratinocytes were exposed to irradiation for 0–170 s using lasers with defined effective power. Using the formula below, the effective laser power, beam diameter, and exposition time were converted to fluence (J/cm^2^). Moreover, due to each laser’s effective power differences, the investigated fluences are different for each wavelength. Finally, the fluence range to be investigated for each laser and cell line was determined using Alamar blue assay (data not shown).

$$Fluence\;\left[\frac{J}{cm^{2}}\right]=\frac{Effective\;power\;\left[W\right]\times Exposition\;time\;\left[s\right]}{Beam\;area\;\left[cm^{2}\right]}$$

## 5. Limitations

Two-dimensional, monolayer cell cultures allow for a broad and reproducible research spectrum. In this study, we investigated the direct effects of laser irradiation on the keratinocytes and fibroblasts using adherent cell cultures. However, 2D cell cultures do not mimic the physiological features of the tissues that limit the cell to cell and cell to ECM interactions [41]. Moreover, laser exposure might also affect the cells in different ways, especially by inducing changes in extracellular matrix (ECM) production. Therefore, further studies investigating the effects of non-ionizing radiation on the skin should involve 3D tissue models. The focus should be given to ECM production and the molecular mechanism underlying laser-induced cell proliferation and cell cycle changes.

## Figures and Tables

**Table 1:** Safe fluences of 445 nm, 520 nm, and 638 nm irradiation for Ker-CT keratinocytes and BJ-5ta.

	BJ-5ta	Ker-CT
445 nm	<59.7 J/cm^2^	>204.1 J/cm^2^
520 nm	<468.4 J/cm^2^	<385.7 J/cm^2^
638 nm	<151.5 J/cm^2^	<151.5 J/cm^2^

**Table 2:** Basic information about laser system.

Wavelength (nm)	Power during Continuous-Wave Operation (mW)	Effective Power (mW)	Beam Diameter (mm)
445	500	200	5
520	1000	600	5
638	700	300	5

## Data Availability

The data presented in this study are available on request from the corresponding author. The data are not publicly available due to founding agreement limitations.

## Supplementary Materials

The following are available online at https://www.mdpi.com/article/10.3390/ijms222111605/s1.
