# Supplementary material for: Effects of 445 nm, 520 nm, and 638 nm Laser Irradiation on the Dermal Cells
Source: Int J Mol Sci. 2021 Oct 27;22(21):11605. doi: 10.3390/ijms222111605 (PMC8584201; doi:10.3390/ijms222111605)
Supplement: Supplementary file 1 [file ijms-22-11605-s001.zip › ijms-1420294-supplementary.pdf]

Detailed data describing viability and death type of KerCT keratinocytes and BJ-5ta fibroblasts exposed to 445 nm, 520 nm and 638 nm irradiation.

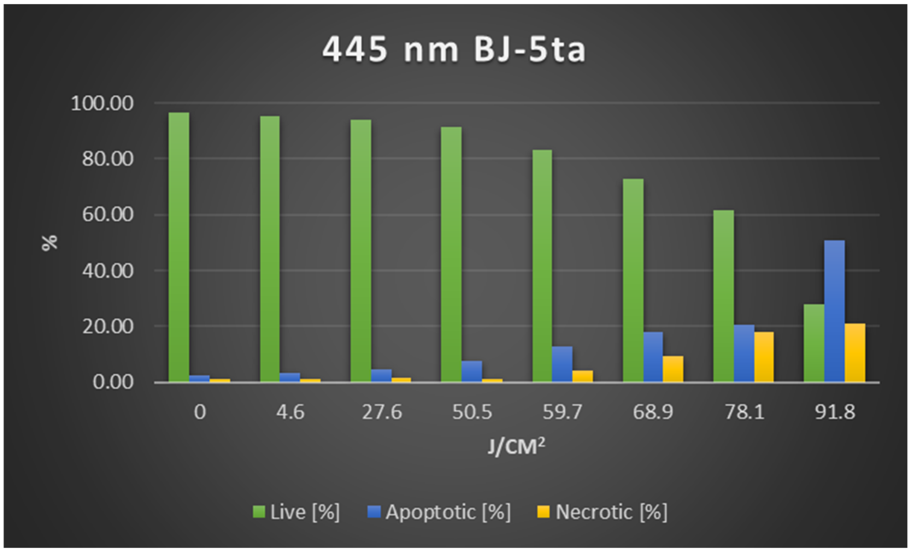

Figure S1. Graphical representation of viability and cell death type of BJ-5ta cells exposed to 445 nm irradiation.

Table S1. Summarized data of viability and cell death type of BJ-5ta cells exposed to 445 nm irradiation.

445 nm BJ-5ta			
**J/Cm²**	**Live [%]**	**Apoptotic [%]**	**Necrotic [%]**
**0**	96.67	2.29	1.04
**4.6**	95.32	3.40	1.28
**27.6**	93.99	4.66	1.35
**50.5**	91.17	7.60	1.23
**59.7**	83.06	12.92	4.02
**68.9**	72.84	17.98	9.17
**78.1**	61.36	20.61	18.03
**91.8**	27.90	50.89	21.16

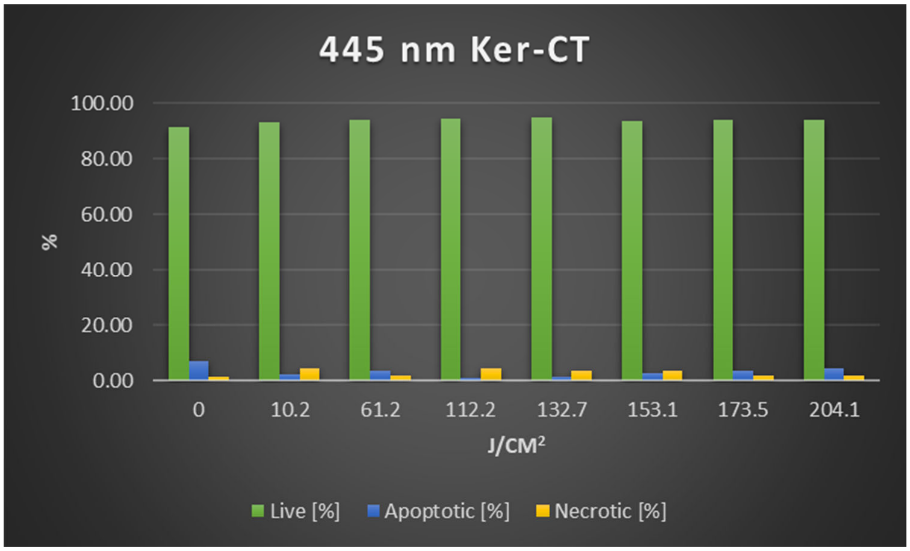

Figure S2. Graphical representation of viability and cell death type of Ker-CT cells exposed to 445 nm irradiation.

Table S2. Summarized data of viability and cell death type of KerCT cells exposed to 445 nm irradiation.

445 nm Ker-CT			
**J/Cm^2**	**Live [%]**	**Apoptotic [%]**	**Necrotic [%]**
**0**	91.45	7.24	1.31
**10.2**	93.17	2.22	4.62
**61.2**	94.11	3.82	2.07
**112.2**	94.40	1.18	4.42
**132.7**	95.02	1.51	3.47
**153.1**	93.65	2.77	3.58
**173.5**	94.31	3.78	1.90
**204.1**	93.88	4.35	1.77

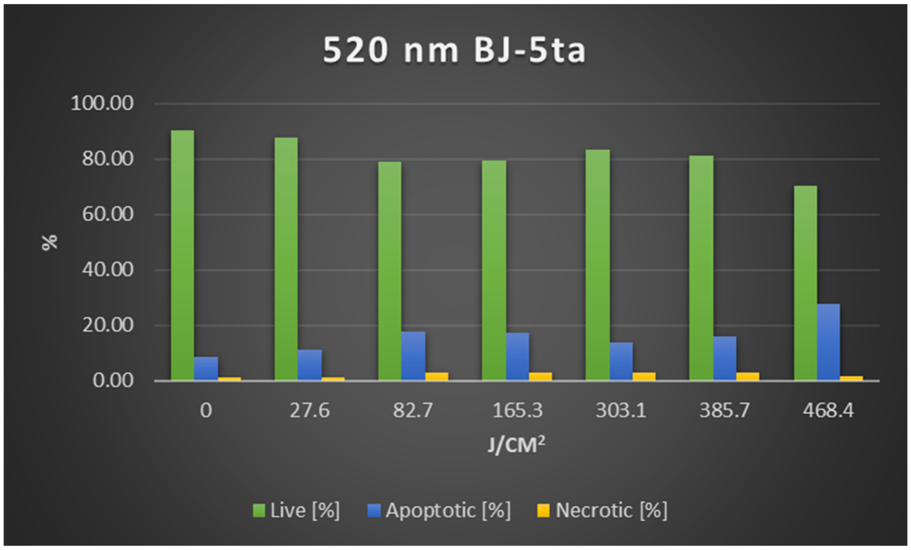

Figure S3. Graphical representation of viability and cell death type of BJ-5ta cells exposed to 520 nm irradiation.

Table S3. Summarized data of viability and cell death type of BJ-5ta cells exposed to 520 nm irradiation.

520 nm BJ-5ta			
**J/Cm^2**	**Live [%]**	**Apoptotic [%]**	**Necrotic [%]**
**0**	90.19	8.59	1.22
**27.6**	87.68	11.15	1.17
**82.7**	78.99	17.87	3.14
**165.3**	79.52	17.41	3.07
**303.1**	83.35	13.74	2.92
**385.7**	81.06	15.95	3.00
**468.4**	70.40	27.86	1.75

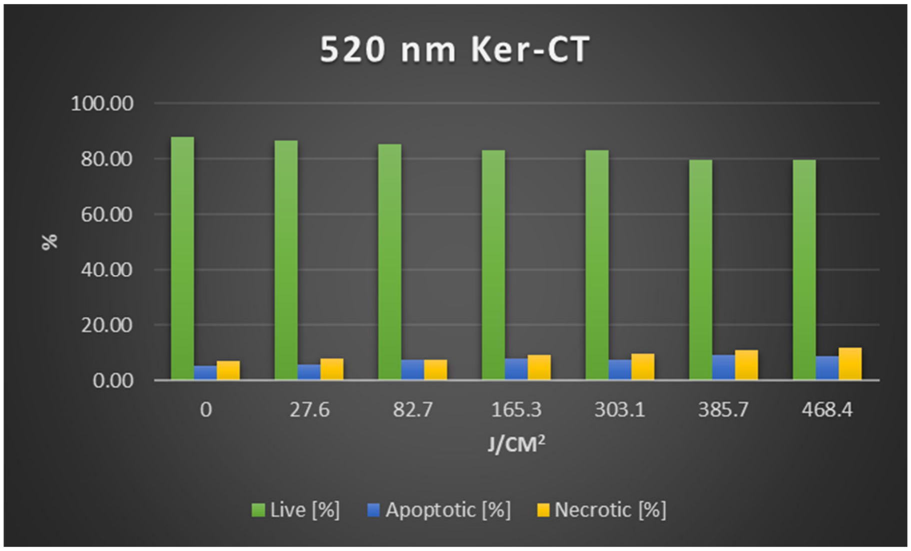

Figure S4. Graphical representation of viability and cell death type of Ker-CT cells exposed to 520 nm irradiation.

Table S4. Summarized data of viability and cell death type of KerCT cells exposed to 520 nm irradiation.

520 nm Ker-CT			
**J/Cm^2**	**Live [%]**	**Apoptotic [%]**	**Necrotic [%]**
**0**	87.65	5.27	7.09
**27.6**	86.57	5.72	7.71
**82.7**	85.24	7.27	7.49
**165.3**	83.10	7.79	9.12
**303.1**	82.94	7.45	9.61
**385.7**	79.75	9.24	11.02
**468.4**	79.44	8.68	11.89

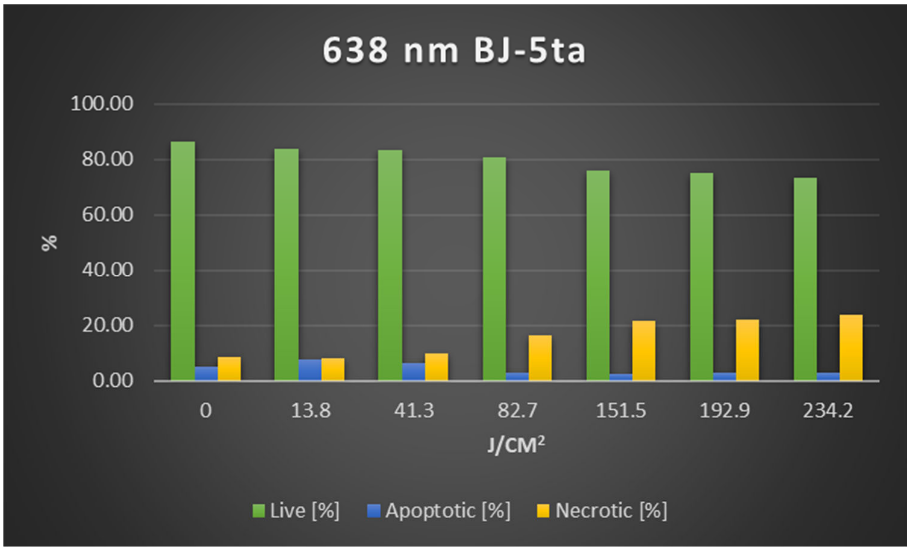

Figure S5. Graphical representation of viability and cell death type of BJ-5ta cells exposed to 638 nm irradiation.

Table S5. Summarized data of viability and cell death type of BJ-5ta cells exposed to 638 nm irradiation.

638 nm BJ-5ta			
**J/Cm^2**	**Live [%]**	**Apoptotic [%]**	**Necrotic [%]**
**0**	86.22	5.07	8.71
**13.8**	83.78	7.84	8.38
**41.3**	83.56	6.53	9.91
**82.7**	80.87	2.80	16.33
**151.5**	75.81	2.43	21.76
**192.9**	74.92	3.02	22.07
**234.2**	73.26	2.80	23.94

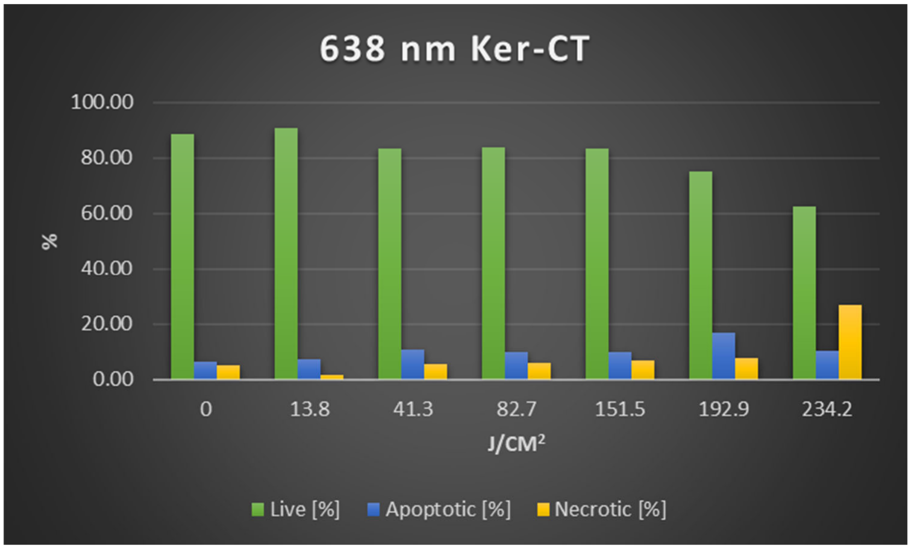

Figure S6. Graphical representation of viability and cell death type of Ker-CT cells exposed to 638 nm irradiation.

Table S6. Summarized data of viability and cell death type of KerCT cells exposed to 638 nm irradiation.

638 nm Ker-CT			
**J/Cm^2**	**Live [%]**	**Apoptotic [%]**	**Necrotic [%]**
**0**	88.71	6.27	5.02
**13.8**	90.97	7.34	1.69
**41.3**	83.43	10.80	5.76
**82.7**	84.05	9.73	6.21
**151.5**	83.28	9.93	6.80
**192.9**	75.32	16.69	7.99
**234.2**	62.60	10.47	26.94
